# Unresectable Hepatocellular Carcinoma: Transcatheter Arterial Chemoembolization Combined With Microwave Ablation vs. Combined With Cryoablation

**DOI:** 10.3389/fonc.2020.01285

**Published:** 2020-08-07

**Authors:** Jialiang Wei, Wei Cui, Wenzhe Fan, Yu Wang, Jiaping Li

**Affiliations:** ^1^Department of Interventional Oncology, The First Affiliated Hospital, Sun Yat-sen University, Guangzhou, China; ^2^Department of Interventional Therapy, Cancer Center, Guangdong Provincial People's Hospital, Guangdong Academy of Medical Sciences, School of Medicine, South China University of Technology, Guangzhou, China

**Keywords:** hepatocellular carcinoma, transcatheter arterial chemoembolization, microwave ablation, cryoablation, combination therapy

## Abstract

**Background:** Transcatheter arterial chemoembolization (TACE) combined with ablation has been widely used for treating unresectable hepatocellular carcinoma (HCC). However, the technique with which TACE should be combined for it to be more effective remains unknown.

**Purpose:** To retrospectively evaluate the efficacy and safety of TACE combined with microwave ablation (MWA) vs. TACE combined with cryoablation (CRA) in treating unresectable HCC.

**Materials and Methods:** From January 2011 to December 2018, 108 patients diagnosed with unresectable HCC were divided into either the TACE-MWA group (*n* = 48) or TACE-CRA group (*n* = 60). Overall survival (OS) and time to progression (TTP) were compared between the two groups. To reduce potential bias, a propensity score matching (PSM) was performed. Complications were observed. Kaplan-Meier survival curves were constructed and compared using the log-rank test.

**Results :** The baseline characteristics of the two groups were balanced. The median OS was 20.9 months (95% CI 14.3–27.6 months) in the TACE-MWA group and 13.0 months (95% CI 8.8–17.1 months) in the TACE-CRA group (*P* = 0.096). The median TTP was 8.8 months (95% CI 4.3–13.4 months) in the TACE-MWA group and 9.3 months (95% CI 7.1–11.5 months) in the TACE-CRA group (*P* = 0.675). After PSM, 48 patients remained in each group. The median OS in the TACE-MWA and TACE-CRA groups was 20.9 months (95% CI 14.3–27.6 months), and 13.5 months (95% CI 8.4–18.6 months, *P* = 0.100), respectively. The median TTP in the TACE-MWA and TACE-CRA groups was 8.8 months (95% CI 4.3–13.4 months), and 8.6 months (95% CI 3.1–14.2 months, *P* = 0.909), respectively. The overall incidence rate of ablation-related complications was lower in the TACE-MWA group than in the TACE-CRA group (66.7 vs. 88.3%, *P* = 0.006). Multivariate analysis showed that the presence of portal vein tumor thrombus (PVTT) and the maximum diameter of intrahepatic tumor were significant prognostic factors for OS and TTP.

**Conclusion:** The efficacy of TACE-MWA and TACE-CRA in the treatment of unresectable HCC was comparable. TACE-MWA was more promising because of a lower complication rate, especially with regard to thrombocytopenia. Further prospective randomized controlled trials are required to validate our findings.

## Introduction

Hepatocellular carcinoma (HCC) has moved upward to become the fourth most common cause of cancer-related death in the world (1, 2). Transcatheter arterial chemoembolization (TACE) is the first-line treatment for patients with unresectable, intermediate-stage HCC, and also effective in patients with advanced-stage HCC (2, 3). However, tumor recurrence and metastasis often occur due to incomplete embolization, tumor neovascularization, the lack of vascular access to the tumor, and difficulties associated with super selective embolization. The long-term efficacy of TACE alone is thus not satisfactory (4–7), and combining other therapies with TACE has become a strategy. TACE combined with ablation therapy, targeted molecular therapy, and radioactive seed implantation have been effective to varying degrees (8–10).

Percutaneous local ablation therapies, such as radiofrequency ablation (RFA), microwave ablation (MWA), and cryoablation (CRA) are recommended in HCC patients with Barcelona Clinic Liver Cancer (BCLC) stage 0 or A who are not candidates for surgery. The main methods employed now are RFA and MWA (2). Previous studies have found that MWA was comparable in efficacy and safety to RFA in treating small and medium-size intrahepatic tumors (11). Relative to RFA and MWA, CRA has an advantage in treating unresectable HCC due to its specific mechanism of action, such as the formation of a visual ice-ball, less damage to the adjacent great blood vessels or organs, less severe pain, and the activation of cyroimmunlogy in tumor (12). A previous study found no significant difference between RFA and CRA in the treatment of stage I and II HCC (13). There are relatively few comparative studies on the treatment of HCC by MWA vs. CRA, especially for large HCC. However, for large unresectable HCC, ablation monotherapy is rarely reported. Combination therapy has become a common treatment strategy to improve local control and decrease distant recurrence, (9).

TACE combined with ablation therapy has been shown to be safe and effective (10). Compared with TACE alone or ablation alone, TACE combined with ablation can significantly improve the efficacy for two specific reasons: (1) after TACE, the blood supply to tumor can be reduced, thereby making ablation more effective; (2) the iodide oil deposited by the TACE procedure can allow guidance during ablation under unenhanced CT scan (14, 15). Previous studies have found that TACE combined with MWA or RFA can prolong the overall survival of patients than TACE alone (6, 8, 16). In large unresectable HCC cases, although there was no significant difference between MWA and RFA in terms of the efficacy and safety, MWA has some advantages, including consistently higher intratumor temperature, faster ablation time, multiple applicators, less heat sink effect and a wider range (17, 18). Our previous study confirmed that TACE combined with CRA can improve overall survival in patients with HCC when compared with TACE alone (12). However, it is not clear whether TACE combined with MWA or TACE combined with CRA is more effective (9, 19–21). In this study, we aim to evaluate comparative differences in the efficacy and safety of TACE combined with MWA and TACE combined with CRA for the treatment of patients with unresectable HCC.

## Materials and Methods

### Study Design

This is a retrospective study from our center. The study was approved by the ethics committee of our hospital and conducted in accordance with the guidelines of the Helsinki declaration. Written informed consent was waived because the study was retrospective.

The study inclusion criteria were as follows: (a) 18–75 years old; (b) newly diagnosed with HCC, according to EASL or AASLD guidelines (3, 22); (c) BCLC stage B or C, without candidacy for surgical resection or transplantation; (d) Child-Pugh class A or B; (e) laboratory tests values (platelet count >60 × 10^9^/L, hemoglobin concentration >85 g/L, prothrombin time elevated >6 s); (f) normal renal function (serum creatinine concentration 1.5 times or lower than the upper limit of the normal range), and (g) a performance status score of 0–2 in the eastern cooperative tumor group (ECOG). We excluded patients exhibiting any of the following: the obstruction of the main portal vein, previous liver resection, as well as a history of liver transplantation, treatments such as radioactive seed implantation, targeted therapy or systemic chemotherapy.

**Figure 1:**
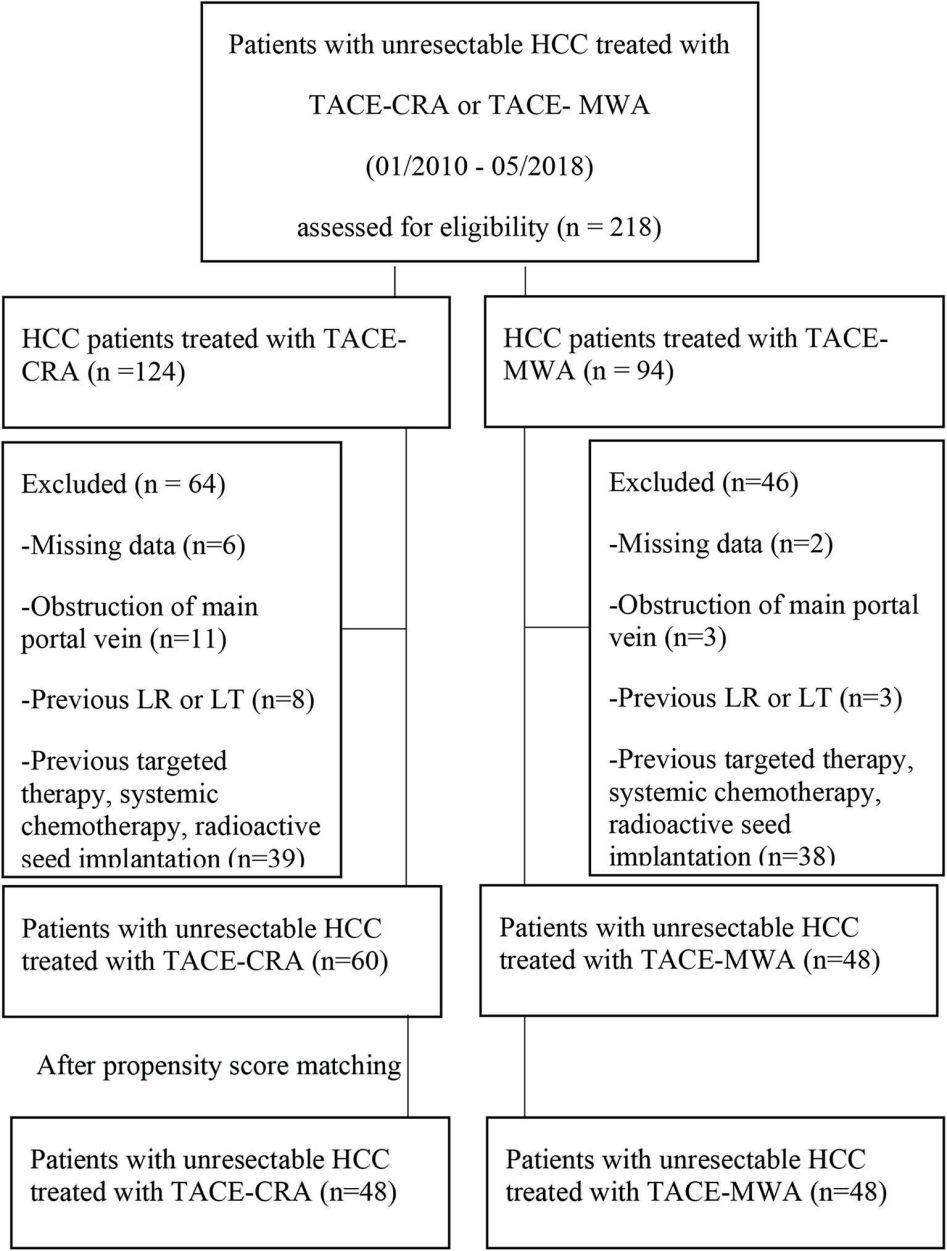
Flowchart shows patients selection. HCC, hepatocellular carcinoma; CRA, cryoablation; MWA, microwave ablation; TACE, transcatheter arterial chemoembolization; LR, liver resection; LT, liver transplantation.

From January 2010 to December 2018, 218 patients received either TACE-MWA or TACE-CRA. A total of 110 patients were excluded (Figure 1). Finally, a total of 108 patients were enrolled, and TACE was the first-line treatment. All patients either had no indication for surgery or refused surgery after multidisciplinary discussion with the same team. All patients were informed of the advantages and disadvantages of MWA and CRA, including expected treatment outcome, treatment-related morbidity, and cost. The choice of ablation modalities was ultimately made by patients and their authorized representatives. Patients were divided into the TACE-MWA group and TACE-CRA group based on the treatment they received.

### TACE Protocol

TACE was performed by three radiologists (JL, YW, and WF), with over 10–20 years of interventional experience. TACE was performed as previously described (23, 24). A selective 5-Fr YASHIRO or RH catheter was briefly introduced, and a visceral angiogram was performed to evaluate hepatic artery supply. Patients received the super selective catheterization of the hepatic artery supplied by the distal tumor with 2.7-F micro-catheter (Progreat; Terumo), and 5–20 ml lipiodol (Lipiodol; Guerbet, Roissy, France) mixed with 20–40 mg epirubicin (Pfizer, Wuxi, China) were slowly injected until the blood flow slowed. Finally, 350–560 μm of polyvinyl alcohol particles (Alicon Pharmaceutical, Hangzhou, China) were injected to reduce tumor blood flow if necessary. All patients received contrast-enhanced dynamic CT or MR imaging within 2 weeks before the first TACE. After the first TACE, ablation was performed within 2 weeks. Tumor response was evaluated with enhanced CT at 4–6 weeks after treatment according to the mRECIST guidelines. Based on the evaluation of results, TACE was given on-demand treatment.

### Percutaneous Microwave Ablation Procedure

If CT or MRI reexamination showed that the intrahepatic lesion was still not regressing after the first TACE, MWA was offered to patients who were not expected to have complete tumor necrosis after a second TACE session. In the TACE-MWA group, percutaneous MWA was generally performed by the same team of doctors in 2 weeks after the first TACE procedure. Most of the patients were under conscious sedation. General anesthesia was reserved for cases in which intra-procedural pain became problematic. MWA was performed using the MTC-3C microwave therapy instruments (Vison-China Medical Devices R&D Institute, Nanjing, China) set to a frequency of 2,450 MHz±10% and an output power of 5–120 W. The microwave antenna was a 15-gauge cooling unipolar needle, either 150 or 180 mm long. MWA output power was 50–80 W applied for 5–10 min per ablation depending on the specific situation. Ablations can lead to coagulation necrosis using microwave less than four antenna to achieve a suitable ablation volume.

### Percutaneous Cryoablation Procedure

Similarly, the efficacy of the first TACE was evaluated, and if the intrahepatic lesion was still not regressing, CRA was offered to patients who were not expected to have complete tumor necrosis after a second TACE session. All percutaneous CRA procedures in the TACE-CRA group were performed by the same team of doctors who performed the first TACE procedure. The CRA procedure was performed using techniques previously described (12). Most of the patients tolerated to the procedure under conscious sedation, although a general anesthetic was used for cases where intra-procedural pain was problematic. CRA is a process that uses extreme cold to destroy or damage tissue (25). Procedures were guided by computed tomography. The CRA procedures were performed using an argon-based cryoablation system (Cryo-Hit, Galil Medical, Yokneam, Israel) and 17-gauge cryoablation applicators. One to five applicators were used to achieve an ice ball that completely encompassed the tumor with a 5 mm or greater margin of uninvolved liver beyond the tumor. The CRA procedure took more than one session in such situations.

### Assessment of Response and Follow-Up Protocol

The primary end point was overall survival (OS), which was defined as the time from the beginning of the first TACE treatment to death or the last follow-up. The secondary endpoints included tumor response and time to tumor progression (TTP), which was defined as the time from the beginning of the first TACE treatment to radiologic tumor progression, death, or the last follow-up. Tumor evaluation indicators included objective response rate (ORR) and disease control rate (DCR), as described in the modified Response Evaluation Criteria in Solid Tumors (mRECIST) (26). ORR refers to the proportion of patients whose tumors shrank to a certain amount and remained unchanged for a certain period of time, including complete response (CR) and partial response (PR) cases. DCR refers to the proportion of patients whose tumors shrank or stabilized for a certain period of time, including CR, PR, and stable disease (SD) cases. Complications were observed clinically during admission and assessed by telephone interview after discharge. They were graded according to the Common Terminology Criteria for Adverse Events (CTCAE, version 3.0) (27).

### Statistical Analysis

All statistical analyses were performed using SPSS software version 23.0 software (IBM SPSS, Chicago, IL). Continuous variables between the two groups were expressed as median ± SD, and compared using the Student's *t*-test. Categorical variables were expressed as percentage and frequency, and compared using the χ^2^ test. Survival curves were constructed using the Kaplan-Meier method and were compared using the log-rank test. Univariate and multivariate analyses with various parameters were performed using Cox's regression model with proportional hazards. The relative prognostic significance of the variables in predicting the overall survival rate and the time to tumor progression rate or metastasis was assessed using multivariate Cox proportional hazards regression analysis and logistic regression analysis, respectively. To minimize the selection bias, a 1:1 propensity score matching (PSM) was performed using the nearest-neighbor matching method with a caliper distance of 0.1 without replacement. All independent variables were entered into the propensity model. All statistical tests were two-tailed, and *P* < 0.05 were considered statistically significant.

## Results

### Patients

The baseline characteristics are presented in Table 1. No significant differences were observed between the TACE-MWA group and the TACE-CRA group before and after PSM (Table 1). In both TACE-MWA and TACE-CRA groups, the median number of the TACE procedure performed was 2 (range 1–7). In TACE-MWA and TACE-CRA groups, the median number of the ablation procedure performed was 2.0 (range 1–3) and 1.0 (range 1–5), respectively.

**Table 1:** Patients characteristics.

	**Overall series**			**Propensity score-matched pairs**		
**Parameter**	**TACE + CRA *N =* 60 (%)**	**TACE + MWA*N =* 48 (%)**	***P*-value**	**TACE + CRA*N =* 48 (%)**	**TACE + MWA*N =* 48 (%)**	***P*-value**
Sex			>0.999			0.726
Female	6 (10.0)	5 (10.4)		4 (8.3)	5 (10.4)	
Male	54 (90.0)	43 (89.6)		44 (91.7)	43 (89.6)	
Age (y), mean ± SD	52.9 ± 11.4	54.7 ± 10.3	0.415	51.9 ± 11.2	54.7 ± 10.3	0.207
≤ 60	38 (63.3)	31 (64.6)	0.893	33 (68.8)	31 (64.6)	0.665
> 60	22 (36.7)	17 (35.4)		15 (31.2)	17 (35.4)	
ECOG PS			0.490			0.305
0	26 (43.3)	24 (50.0)		19 (39.6)	24 (50.0)	
1	34 (56.7)	24 (50.0)		29 (60.4)	24 (50.0)	
HBsAg			>0.999			0.726
Positive	54 (90.0)	44 (91.7)		43 (89.6)	44 (91.7)	
Negative	6 (10.0)	4 (8.3)		5 (10.4)	4 (8.3)	
HCV			>0.999			0.557
Positive	2 (3.3)	1 (2.1)		2 (4.2)	1 (2.1)	
Negative	58 (96.7)	47 (97.9)		46 (95.8)	47 (97.9)	
Cirrhosis			0.314			0.529
Yes	33 (55.0)	31 (64.6)		28 (58.3)	31 (64.6)	
No	27 (45.0)	17 (35.4)		20 (41.7)	17 (35.4)	
Ascites			0.915			0.805
Yes	12 (20.0)	10 (20.8)		11 (22.9)	10 (20.8)	
No	48 (80.0)	38 (79.2)		37 (77.1)	38 (79.2)	
Tumor diameter (cm)	11.8 ± 5.3	11.5 ± 4.9	0.748	12.4 ± 5.5	11.5 ± 4.9	0.403
> 10	39 (65.0)	26 (54.2)	0.253	33 (68.8)	26 (54.2)	0.142
≤ 10	21 (35.0)	22 (45.8)		15 (31.2)	22 (45.8)	
No. of tumors			0.341			0.660
Solitary	15 (25)	16 (33.3)		14 (29.2)	16 (33.3)	
Multiple	45 (75)	32 (66.7)		34 (70.8)	32 (66.7)	
Tumor growth pattern			0.139			0.138
With capsule	18 (30)	21 (43.7)		14 (29.2)	21 (43.7)	
Infiltrative	42 (70)	27 (56.3)		34 (70.8)	27 (56.3)	
AFP level (ng/ml)			0.762			0.539
≤ 400	32 (53.3)	27 (56.3)		24 (50.0)	27 (56.3)	
> 400	28 (46.7)	21 (43.7)		24 (50.0)	21 (43.7)	
PVTT statue			0.861			0.681
Yes	24 (40.0)	20 (41.7)		22 (45.8)	20 (41.7)	
No	36 (60.0)	28 (58.3)		26 (54.2)	28 (58.3)	

*ECOG PS, eastern cooperative oncology group performance status; HBsAg, hepatitis B surface antigen; HCV, hepatitis C virus; PVTT, portal vein tumor thrombosis; AFP, α-Fetoprotein; HCC, hepatocellular carcinoma*.

### Tumor Response

Six patients (12.5%) in the TACE-MWA group vs. 4 patients (6.7%) in the TACE-CRA group had a CR, while 29 patients (60.8%) in the TACE-MWA group vs. 16 patients (26.7%) in the TACE-CRA group had a PR. 35 patients (60.4%) in the TACE-MWA group vs. 20 patients (33.3%) in the TACE-CRA group achieved an objective response (*P* = 0.161). 44 patients (91.7%) in the TACE-MWA group vs. 45 patients (75%) in the TACE-CRA group achieved disease control (*P* < 0.001). Additionally, 15 patients (25%) in the TACE-CRA group and 4 patients (8.3%) in the TACE-MWA group had a PD.

### Complications

No unexpected treatment-related deaths were observed. Complications after ablation therapy and TACE are shown in Tables 2, 3. The most common complications after ablation were fever, abdominal pain, local skin frostbite, hemorrhage, and thrombocytopenia. The most common grade 1–2 complications were abdominal pain and thrombocytopenia in the TACE-CRA group. Three patients suffered from local skin frostbite in the TACE-CRA group. Four patients in the TACE-CRA group who suffered from grade 3–4 thrombocytopenia were treated with recombinant human interleukin-11 to assist with recovery. One patient developed a liver abscess after CRA. New ascites appeared in another patient after CRA. No cryoshock, liver failure, pneumonia, acute myocardial infarction, hepatorenal syndrome, or other severe complication happened in either group after ablation therapy.

**Table 2:** Complications related to CRA/MWA in the two group.

**Complications**	**TACE-CRA (*****N = 60)***		**TACE-MWA (*****N = 48)***		***P*****-Value**	
	**Any grade (%)**	**Grade 3–4 (%)**	**Any grade (%)**	**Grade 3–4 (%)**	**Any grade (%)**	**Grade 3–4 (%)**
Overall incidence	53 (88.3)	7 (11.7)	32 (66.7)	2 (4.2)	0.006	0.293
Fever	7 (11.7)	0	5 (10.4)	0	0.837	…
Abdominal pain	38 (70.0)	4 (6.7)	25 (52.1)	2 (4.2)	0.057	0.888
Frostbite/burns	3 (5.0)	0	0	0	0.326	…
Pleural effusion	4 (6.7)	0	0	0	0.190	…
Hemorrhage	1 (1.7)	0	0	0	…	…
Thrombocytopenia	25 (41.7)	4 (6.7)	4 (8.3)	0	0.000	0.190
New ascites	1 (1.7)	0	0	0	…	…
Liver abscess	1 (1.7)	0	0	0	…	…

**Table 3:** Complications related to TACE in the two group.

**Complications**	**TACE-CRA (*N =* 60, %)**	**TACE-MWA (*N =* 48, %)**	***P*-Value**
Overall incidence	45 (75%)	38 (79.2)	0.610
Fever,	35 (58.3)	22 (45.8)	0.259
Abdominal pain, grade 1–2	39 (65%)	19 (39.6)	0.348
Nausea/vomiting, grade 1–2	6 (10)	9 (18.6)	0.010
New ascites	5 (8.3)	0	…
Liver dysfunction, grade 1–2	0	1 (2.1)	…
Pleural effusion	0	1 (2.1)	…

### Survival

**Figure 2:**
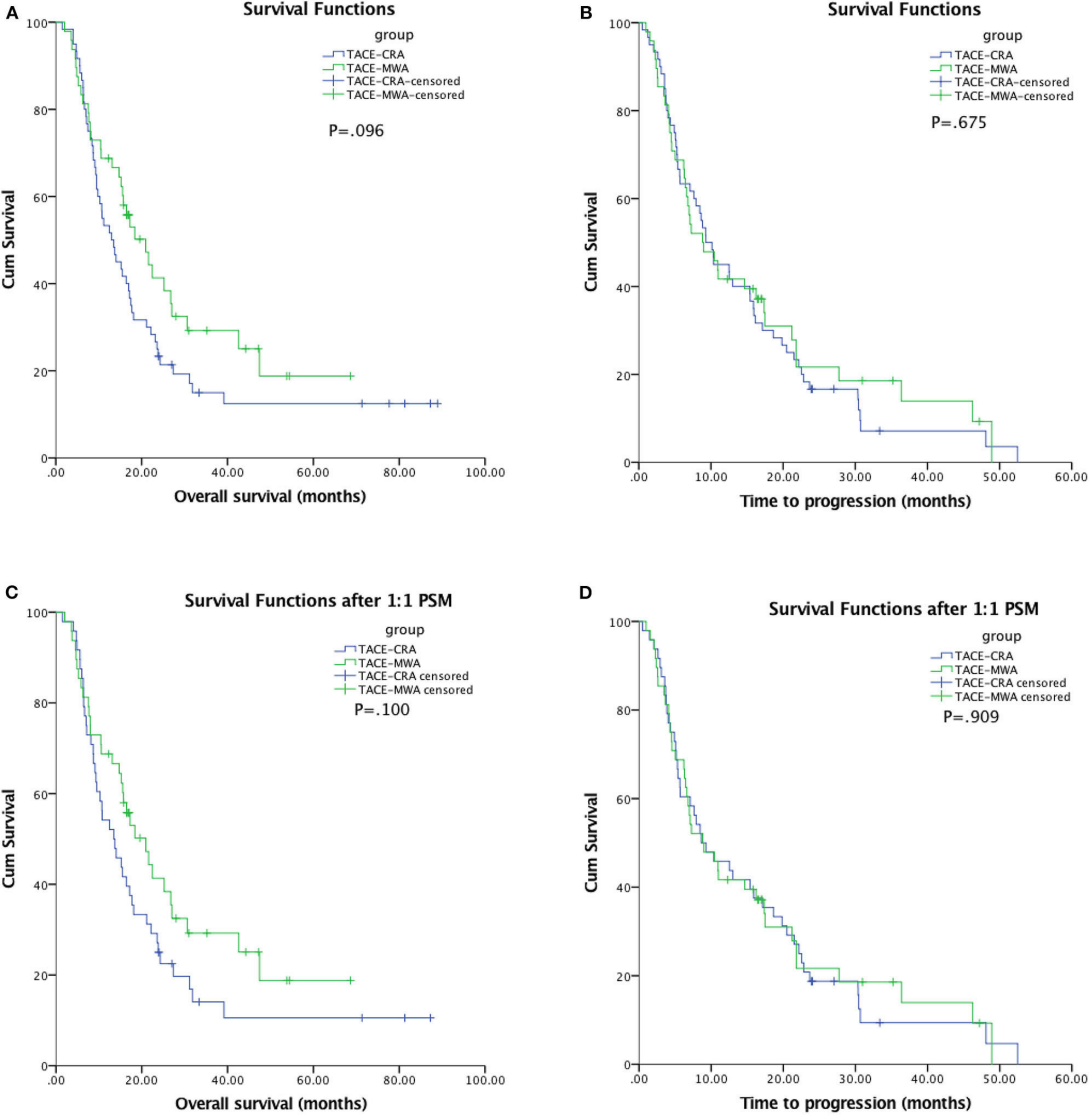
**(A)** Kaplan-Meier curves of overall survival (OS) in patients with hepatocellular carcinoma treated with TACE-MWA (*n* = 48; median OS, 20.9 months) and TACE-CRA (*n* = 60; median OS, 13.0 months; *P* = 0.096). **(B)** Kaplan-Meier curves of time to progression (TTP) in patients with hepatocellular carcinoma treated with TACE-MWA (*n* = 48; median TTP, 8.8 months) and TACE-CRA (*n* = 60; median TTP, 9.3 months; *P* = 0.675). **(C)** Kaplan-Meier curves of overall survival (OS) in patients with hepatocellular carcinoma treated with TACE-MWA (*n* = 48; median OS, 20.9 months) and TACE-CRA (*n* = 48; median OS, 13.5 months; *P* = 0.100). **(D)** Kaplan-Meier curves of time to progression (TTP) in patients with hepatocellular carcinoma treated with TACE-MWA (*n* = 48; median TTP, 8.8 months) and TACE-CRA (*n* = 48; median TTP, 8.6 months; *P* = 0.909).

**Figure 3:**
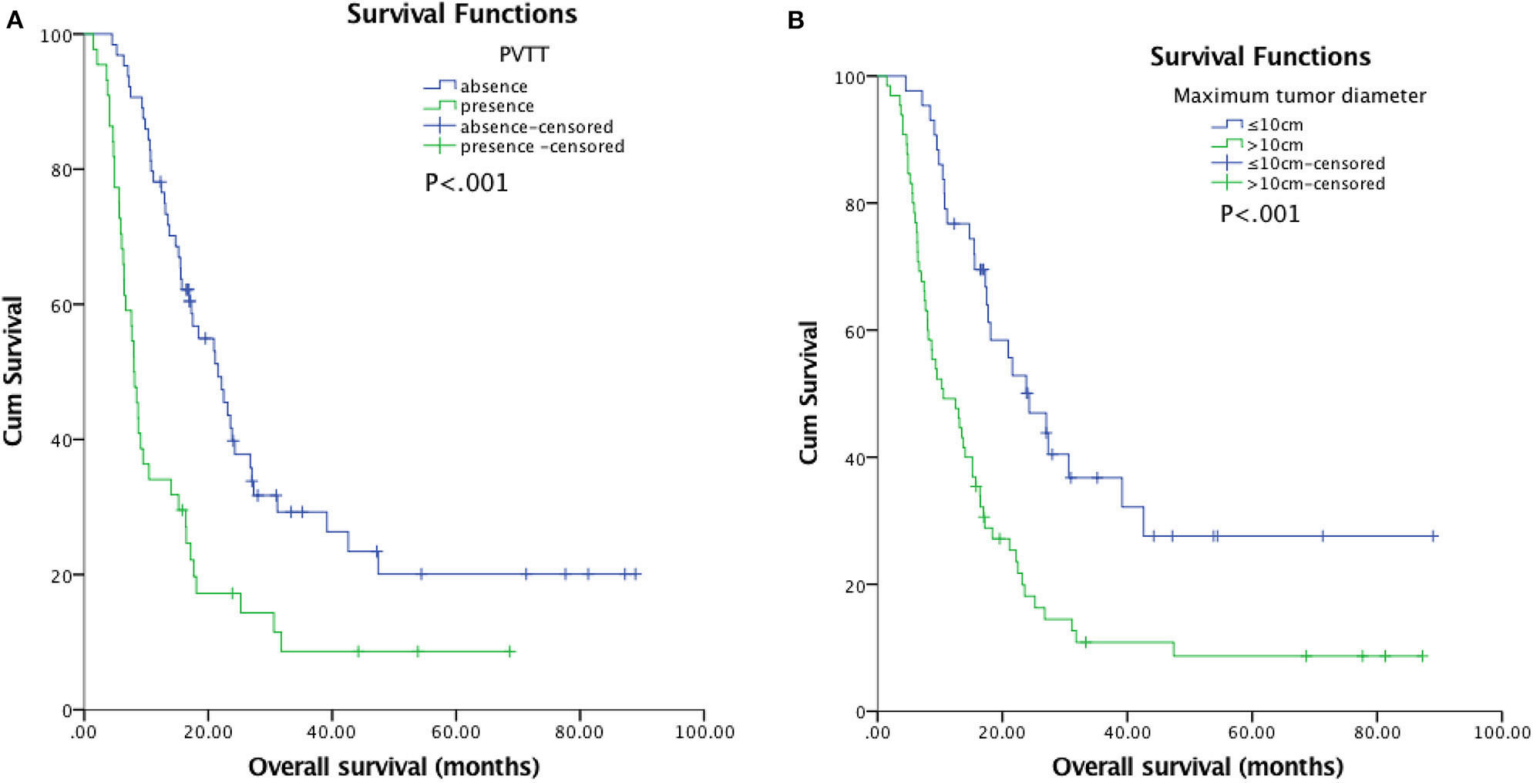
**(A)** Kaplan-Meier curves of overall survival (OS) in patients with hepatocellular carcinoma with presence of PVTT (*n* = 44; median OS, 8.0 months) and absence of PVTT (*n* = 64; median OS, 21.6 months; *P* < 0.001). However, multivariable analysis showed that the difference had significant between the two groups (*P* = 0.016). **(B)** Kaplan-Meier curves of overall survival (OS) in patients with hepatocellular carcinoma with a maximum tumor diameter ≤10 cm (*n* = 43; median OS, 24.3 months) or >10 cm (*n* = 65; median OS, 10.6 months; *P* < 0.001). However, multivariable analysis showed that the difference had significant between the two groups (*P* = 0.006).

The median follow-up duration was 77.7 months (95% CI: 22.7, 132.6 months). At the last follow-up, 16 patients in TACE-MWA group and 9 patients in TACE-CRA group were still alive. The median OS was 20.9 months (95% CI 14.3–27.6 months) in TACE-MWA group, and 13.0 months (95% CI 8.8–17.1 months) in TACE-CRA group (*P* = 0.096). The median TTP was 8.8 months (95% CI 4.3–13.4 months) in the TACE-MWA group, and 9.3 months (95% CI 7.1–11.5 months) in the TACE-CRA group (*P* = 0.675). There was no statistically significant difference in OS or TTP between the two groups (Figures 2A,B). After 1:1 PSM, 48 patients remained in each group. All variables were matched between the two groups (Table 1). The median OS in the TACE-MWA and TACE-CRA groups was 20.9 months (95% CI 14.3–27.6 months), and 13.5 months (95% CI 8.4–18.6 months, *P* = 0.100), respectively. The median TTP in the TACE-MWA and TACE-CRA groups was 8.8 months (95% CI 4.3–13.4 months), and 8.6 months (95% CI 3.1–14.2 months, *P* = 0.909), respectively (Figures 2C,D). Univariate analysis showed that the presence of ascites, presence of portal vein tumor thrombus (PVTT), maximum diameter of intrahepatic tumor, tumor growth pattern, and α-fetoprotein level were associated with OS and TTP (*P* < 0.05) (Tables 4, 5). Multivariate analysis showed that the presence of PVTT and the maximum diameter of intrahepatic tumor were statistically significant prognostic factors for OS and TTP (*P* < 0.05) (Tables 4, 5; Figures 3A,B).

**Table 4:** Univariate and multivariate analysis of predictors of OS.

**Factor**	**Univariate analysis**		**Multivariate analysis**	
	**HR**	***P*-value**	**HR**	***P-*value**
Group (TACE-CRA vs. TACE-MWA)	0.688 (0.442, 1.072)	0.099	…	…
Sex (male vs. female)	1.356 (0.625, 2.944)	0.441	…	…
Age (>60 vs. ≤ 60 y)	0.631 (0.400, 0.997)	0.049	0.928 (0.521, 1.653)	0.801
ECOG (1 vs. 0)	1.018 (0.661, 1.567)	0.937	…	…
HBsAg (positivity vs. negativity)	0.937 (0.451, 1.944)	0.861	…	…
Cirrhosis (Presence vs. absence)	1.458 (0.928, 2.291)	0.102	…	…
Ascites (Presence vs. absence)	2.132 (1.277, 3.559)	0.004	1.414 (0.751, 2.662)	0.283
PVTT (Presence vs. absence)	2.449 (1.584, 3.785)	0.000	1.928 (1.113, 3.282)	0.016
Maximum diameter of intrahepatic tumor (>10 vs. ≤ 10 cm)	2.414 (1.513, 3.851)	0.000	2.020 (1.225, 3.331)	0.006
Tumor growth pattern (infiltrative vs. with capsule)	1.634 (1.026, 2.602)	0.039	11.259 (0.748, 2.120)	0.386
No. classification of intrahepatic tumor (multiple vs. solitary)	0.638 (0.389, 1.048)	0.076	…	…
α-Fetoprotein (> 400 vs. ≤ 400 ng/ml)	1.994 (1.291, 3.081)	0.002	1.602 (0.988, 2.599)	0.056

**Table 5:** Univariate and multivariate analysis of predictors of TTP.

**Factor**	**Univariate analysis**		**Multivariate analysis**	
	**HR**	***P*-value**	**HR**	***P*-value**
Group (TACE-CRA vs. TACE-MWA)	0.916 (0.606, 1.383)	0.676	0.948 (0.606, 1.483)	0.815
Sex (male vs. female)	1.228 (0.615, 2.456)	0.560	…	…
Age (> 60 vs. ≤ 60 y)	0.733 (0.477, 1.126)	0.156	…	…
ECOG (1 vs. 0)	1.306 (0.864, 1.974)	0.205	…	…
HBsAg (positivity vs. negativity)	0.660 (0.341, 1.280)	0.219	…	…
Cirrhosis (Presence vs. absence)	1.454 (0.948, 2.228)	0.086	…	…
Ascites (Presence vs. absence)	2.287 (1.388, 3.769)	0.001	1.431 (0.759, 2.698)	0.268
PVTT (Presence vs. absence)	2.219 (1.465, 3.360)	0.000	1.650 (1.007, 2.704)	0.047
Maximum diameter of intrahepatic tumor (>10 vs. ≤ 10 cm)	2.551 (1.646, 3.953)	0.000	2.111 (1.308, 3.407)	0.002
Tumor growth pattern (infiltrative vs. with capsule)	1.737 (1.115, 2.706)	0.015	1.418 (0.871, 2.310)	0.160
No. classification of intrahepatic tumor (multiple vs. solitary)	0.727 (0.458, 1.153)	0.176	…	…
α-Fetoprotein (> 400 vs. ≤ 400 ng/ml)	1.717 (1.139, 2.587)	0.010	1.379 (0.851, 2.237)	0.192

## Discussion

In this study, we first evaluated the safety and efficacy of treating unresectable HCC with TACE-MWA vs. TACE-CRA. There were no statistically significant differences in the median OS (20.9 vs. 13.0 months, *P* = 0.096) and median TTP (8.8 vs. 9.3 months, *P* = 0.675) between the TACE-MWA and TACE-CRA groups, respectively; however, our results showed that MWA has fewer complications than CRA in treating unresectable HCC.

Many previous studies have demonstrated that combination therapy is significantly more effective in patients with unresectable HCC (8, 9, 12, 20, 28–30). Ginsburg et al. (31) found that a median OS of TACE plus MWA of about 42.6 months, and complete local tumor response rate was 76.6% (49 of 64 tumors). Ginsburg et al. (31) also inferred that the BCLC stage was associated with OS. Zheng et al. (8) showed that the median TTP and OS of TACE-MWA were 12.5 and 26.6 months, respectively, and that tumor size and number were associated with TTP and OS. Ni et al. (32) obtained a median OS for TACE plus MWA was 21.5 months. In our study, we found that the median TTP and OS of patients in the TACE-MWA group were 8.8 and 18.4 months, respectively, and the local tumor response rate was significantly lower than that in Ginsburg et al. (31). We think the main reason is the huge difference in tumor size between the two studies. In Zheng et al. (8) the average tumor size in the TACE-MWA group was similar to that observed in our study, and the TTP and OS were slightly longer; however, there was no description of tumor capsule and the ECOG score. In our study, the OS of patients in the TACE-MWA group was similar to that observed in Ni et al. (32) however, 67 (77.9%) patients in that study had no portal vein invasion.

Xu et al. (33) divided patients into the TACE-CRA group and CRA alone group, and found that the size and number of tumors in the TACE-CRA group were larger than those in the CRA alone group before treatment. Nevertheless, the 4- and 5-year survival rates of patients in the TACE-CRA group were higher than those in the CRA alone group. Huang Chen et al. (34) designed a prospective study to evaluate the effectiveness of TACE combined with cryoablation vs. TACE alone, and discovered that the complete remission rate and total effective rate of the combination group were significantly higher than those of the TACE group. The aforementioned studies showed that TACE combined with CRA could bring added benefits to patients with unresectable HCC. The effect of combined treatment was significantly better than that of TACE or CRA alone, and no major complications occurred.

In previous studies, some scholars believed that CRA reduced local tumor progression (35) and was suitable for patients with large intrahepatic tumor diameter (29, 33). The maximum diameter of intrahepatic tumors in the TACE-CRA group was larger than that in the TACE-MWA group. Although there was no significant difference, the diameter of tumor was still closely related to survival time (12). Additionally, there was no statistically significant difference in ORR between the two groups; however, DCR in the TACE-MWA group was significantly higher than in the TACE-CRA group. Thus, we believe that TACE-MWA can produce a relatively good outcome in selected patients.

We recorded no mortality in either group, similar to a multicenter Italian study showing that microwave ablation is associated with a low rate of major complications (36). The most common complication after ablation in both groups was abdominal pain, which is considered a common symptom of post-ablation syndrome. In the TACE-CRA group, 3 patients (5%) suffered local skin frostbite, which gradually recovered after rewarming. This underscores the importance of protecting adjacent skin during cryoablation. In our center, we used 1–2 sterile rubber gloves filled with warm water to wrap around the ablation needle puncture site to avoid frostbite. One patient who developed an abscess was treated successfully with ultrasound-guided catheterization and drainage of the hepatic abscess, repeated drainage tube flushing, and anti-infection treatment 2 weeks after CRA. After CRA, one patient suffered from hypoproteinemia and a small amount of ascites, which resolved after intensive nutritional support treatment and the infusion of human blood albumin injection. The ascites was attributed to liver dysfunction secondary to the ablation procedure. In the TACE-CRA group, four patients developed serious thrombocytopenia. This is significantly higher than the TACE-MWA group, and in concordance with the results of a previous study (21). However, platelets returned to normal range after platelet-raising therapy, including platelet infusion and recombinant human interleukin-11.

Our study has some limitations. First, it was a single-center, retrospective study, and there was a selection bias. Some patients refused surgery even after discussion with multidisciplinary team, which could have influenced the results of this study. Although we had applied PSM, selection bias was still unavoidable. Second, the number of patients in the two groups was relatively small. Other disadvantages exist in the study design as well. Although there was no significant difference in baseline data between the two groups, some degree of selection bias was unavoidable. Well-designed, multicenter randomized controlled trials are needed to determine the long-term safety and effects of TACE-MWA and TACE-CRA in treating HCC patients that are deemed unresectable at the time of initial diagnosis.

In conclusion, TACE-MWA and TACE-CRA appear to have equal efficacy in the treatment of unresectable HCC, with TACE-MWA having the added benefit of causing fewer complications in selected patients.

## Data Availability Statement

The datasets analyzed in this article are not publicly available. Requests to access the datasets should be directed to Jiaping Li, jiapingli3s@126.com.

## Ethics Statement

The study involving patients with unresectable hepatocellular carcinoma was reviewed and approved by the ethics committee of the First Affiliated Hospital, Sun Yat-sen University. Written informed consent was waived because the study was retrospective.

## Author Contributions

JL and WC: study conception and design. JW, WC, and WF: data collection and data analysis and interpretation. JW, WC, YW, and WF: manuscript writing and manuscript revise. All authors: manuscript review and final approval of manuscript.

## Conflict of Interest

The authors declare that the research was conducted in the absence of any commercial or financial relationships that could be construed as a potential conflict of interest.
